# Medicine and diet, a common purpose for human health

**Published:** 2008-02-25

**Authors:** Wegnez Léon F.

**Affiliations:** * Secretary General of International Association of Distribution (Association internationale de la distribution) (A.I.D.A.) Director of Royal Belgian Comittee of Distribution (Comité Royal Belge de la Distribution (C.R.B.D).) Member of the French Academy of commercial sciences

**Figure:**
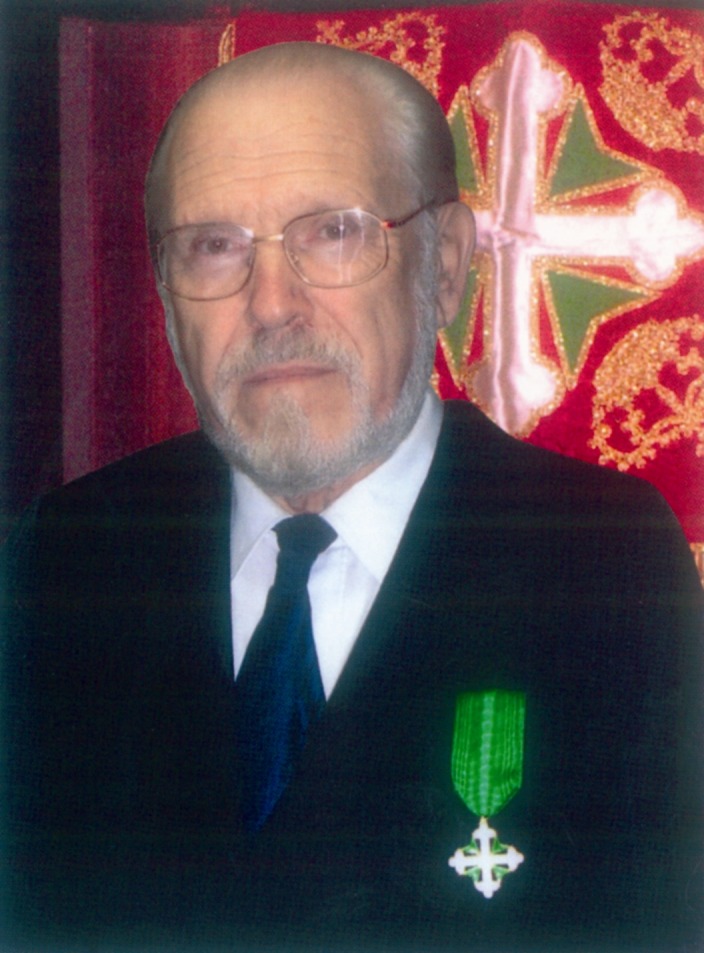


The quality of dietary products and the influence they have on people’s health in the entire world, both man or woman, are the main concern of preoccupation of all conscious instances of populational wellbeing, such as medical world and its patients, various categories of enterprises of production and distribution and their clients and also a multitude of public or private institutions that have as purpose citizen’s protection of interests.

In this context, the International Association of distribution presents the human health issue for fifty years, in 56 adherent countries that are enlisted regularly in its congress schedule and invites great specialists to contribute to a proper human nutrition for the population’s health. It was the subject of the 32nd international congress of A.I.D.A which took place in Parma on the theme of new imperatives of food distribution, a congress organized in close collaboration with the European Food Safety Authority.

We point out the following principles:

a. It is more than ever necessary to establish rigorous systems which guarantee food safety, starting with the production of food and ending with its consumption; this implies that food enterprises must, without a doubt, find a correct balance between their financial needs and their responsibility towards society which includes an effective protection of customers.

b. It is also imperative to ensure a food education in our populations and this is the obligation of the medical world and also of public authorities, of the organisations of consumer protection and of those enterprises which sell their food products in the market and which must associate in this path a clear, objective, true and credible communication to demonstrate their real involvement in ensuring their clients’ health. 

c. In the quest of protecting population’s health, there is a true obligation that must be taken into account by food channels because there is a mutual responsibility of all concerned operators at different levels. One aspect of most priority is to well assure a degree of conformity of each product with legal provisions concerning food quality.

d. As far as trace is concerned, it must be possible to identify the problem in the shortest period of time, downstream to upstream and just as fast to resolve it from upstream to downstream. A good follow-up of batches must allow a very quick intervention in order to withdraw all concerned products and at the same time issue a statement in explicit manner informing consumers about the nature of problems and the measures taken to protect their health.

e. Beyond food composition and food preparation, food preservation is at the very core of the problem. Certainly, the risks that food industry takes are many and of various types but in reality they are frequently made of biological risks. Studies showed that only 3% of food problems encountered by consumers in European countries are attributed to industry, these consumers having most frequently a responsibility in this field by lacking respect for adequate hygiene measures. 

e. All hygiene policies must be founded of the four following principles: precaution, prevention, evaluation of risk and responsibility. It is especially important that the measures taken within the precaution principle be proportional with the risk seriousness. But we will take into account that many families of micro-contaminants endanger human nutrition in a cumulative way, also knowing that certain soil contaminations are prone to last many years.

f. a question of allergies is also raised but because of crossed contamination, food products can have perverse effects on human health therefore we must consider that a few of these effects are real allergies. Some people have a hyper sensibility to certain aliments, but it can be the case of susceptible intolerance with different levels of gravity without being actual allergies. The allergy in fact, is a result of an inopportune reaction of the human organism.

g. At the same time, we must not underestimate the problem of allergies which is a true emerging issue at a public health level. We believe that globally, 30% of individuals are allergic to peanuts, 20% to wheat, 20% to soy, 10% to milk or another 8% to eggs. This is why it is essential to mention on the food wrapping the allergens that the product contains. The state of being allergic may sensibly vary from one individual to another and, in some cases, may be a cause of death.

h. A wide enquiry showed that in 60% of medically documented allergies, the resulting risks of the food product were not mentioned on its pack. The same enquiry showed that at the origin of food allergies in 18% of cases, crossed contaminations were involved and that in 18% of cases there was a mistake in the product’s process of fabrication. 

i. On the other hand, metabolic diseases like obesity and diabetes have become a true health problem at a global populational level. The necessity of taking into consideration the energetic balance, which evens out energetic contribution, through consumed foods in the human organisms with energetic loss, a result of basal metabolism and of lost energy during physical activity -, must be translated into a judicial consumption of nutrients, lipids, glucose and proteins, which will be a true fuel for the human organism.

j. Obesity is a real disease which comprises a variety of risks associated with serious pathologies: hypertension, high levels of serum cholesterol, cardiovascular events, diabetes and respiratory problems as well as some types of cancer. How can the obesity phenomenon that takes truly frightening proportions within different age ranges of our country’s population can be clinically explained? We know that our brain burns glucose only and it is constantly informed of the body’s energetic status. It is the brain then the one that puts into motion our perception of hunger but also on the contrary of satiety. This means that if the brain loses its ability of emitting the satiety signal, an excess of food absorption and uptake will result as well as its consequence, obesity.

k. To fight against obesity is in reality a battle which must be undertaken by both medicine and nutrition. An obese individual has a life expectancy lowered by 10 to 15 years. Among others, we note that 85% of diabetics are overweight or obese, but a widely spread idea must be put aside: it is not obesity that causes diabetes but the other way round. In order to avoid these pathologies without the help of drugs, we must diversify the food that we consume, have an adequate physical activity and good life hygiene and avoid an excess intake of nutrients. 

l. The problem of food wrappings and their being adequate for the products they protect is also insufficiently taken into consideration. At the same time, we can not ignore some food contaminations that result from toxicological migration from the wrapping to its content and the resulting alteration in properties like the taste, smell or colour of the product. The wrapping must ensure the preservation of food product integrity and the principle of optimal precaution is imposed at this level.

m. A special consideration deserves as well the connection between nutrition of children and their growth. Microbiological and chemical risks that affect foods translate into human absorption of toxic substances that will have cumulative negative effect on the health of these children, which are not little adults, but physiologically fragile individuals whose physical build is constantly evolving, especially concerning their digestive system, hepatic and motor mechanisms and immune system. 

n. This is why paediatricians must bring a particular interest into child nutrition and also preoccupy on the future born infant’s nourishment because toxic products inside the body of its mother are susceptible to induce an accumulation of toxins in the baby she is carrying. We will notice, on the other hand, that while a rich maternal milk allows babies to avoid the absorption of a certain number of toxic substances, another milk will expose him, can also be modified by various causes, like weight excess, the cigarette, inadequate diet, mother’s age, her consumption of unhealthy categories of foods or inadequate because she is on a weight loss diet. The presence of pesticides in some food products is susceptible to alter a healthy development of small children. That is why, parent sensitivity and vigilance of paediatricians are imposed, as well as that of gynaecologists which need to protect the health of the mother and her child to be born.

The battle for human health will be won on both medicine and food ground. It is mobilizing for all partners of the great human adventure and appeals decisively to all synergies that justify the fundamental principle of human kind’s most precious asset: health.

